# Primary Small Cell Carcinoma of the Bladder

**DOI:** 10.7759/cureus.15146

**Published:** 2021-05-21

**Authors:** Juliette M Kassas, Julia V Fiuk, Carol A Brenner

**Affiliations:** 1 College of Osteopathic Medicine, University of New England, Biddeford, USA; 2 Urology, Lakes Region General Hospital, Laconia, USA

**Keywords:** small cell carcinomas, non-urothelial bladder cancer, metastatic carcinoma, palliative radiation therapy, chemotherapy

## Abstract

A 64-year-old Caucasian man with a 20 to 25-pack-year cigarette smoking history presented to his primary care provider with the chief complaint of gross hematuria after experiencing three to four months of urinary frequency and urgency. His workup consisted of laboratory blood work, a renal/bladder ultrasound (US), a CT scan without contrast, cystoscopy with biopsy (with an attempted transurethral resection of bladder tumor), and a PET scan. He was diagnosed with stage T4 small cell carcinoma of the bladder (SCCB) shortly after seeking medical care with metastases to the liver, bone, and lymph nodes. There was no evidence of lung involvement. The patient's primary concerns included difficulty urinating and sustained hematuria. He underwent palliative radiotherapy and placement of bilateral nephrostomy tubes in order to preserve his quality of life. He also received a chemotherapy regimen consisting of cisplatin, etoposide, and atezolizumab. The patient underwent hospice care and died approximately six months after the presentation.

## Introduction

Bladder cancer subtypes are differentiated by cellular composition. Urothelial carcinoma (UC), previously known as transitional cell carcinoma, accounts for more than 90% of cases [1]. Squamous cell carcinoma and adenocarcinoma account for up to 5% and 2% of cases, respectively [2-4]. Sarcomas and small cell carcinomas make up the remaining percentage. One source estimated that small cell carcinoma of the bladder (SCCB) accounts for less than 0.7% of all urinary bladder tumors [5]. SCCB tends to be much more aggressive than other bladder tumors with a poor survival outcome. Over 60% of patients have metastatic disease at the time of diagnosis [6]. SCCB has an estimated annual incidence of less than 1-9 per 1 million individuals, with fewer than 1,000 cases identified since 1980 as of 2011 [7]. These statistics were reconfirmed in 2017 [8]. The majority of individuals affected are male (5:1 sex ratio) and Caucasian. The mean age at diagnosis is 67 years and a history of smoking is present in 65-79% of cases [7]. Similar to UC, SCCB most commonly presents as gross hematuria. This is the primary symptom in 63-88% of diagnosed cases [7]. SCCB characteristically co-occurs with other subtypes of bladder cancer with some studies estimating the rate of co-occurrence with UC to be approximately 50-74% [8,9]. Hypotheses exist stating that UC and SCCB likely have common cells of origin or “clone origins,” which further support why they commonly coexist when found [10].

Characterization of cellular tumor subtype is confirmed through tissue visualization and immunohistochemistry staining. Synaptophysin positive staining appears in 72.4% of documented cases, while chromogranin positive staining appears in 50% of patient cases [7]. CD56 positivity serves as a marker of neural cell lineage; it is a feature of small cell carcinoma of any tissue type [11]. Hematoxylin and Eosin (H&E) stain allows for visualization of the affected tissue. Standard features of small cell carcinoma include a large nucleus to cytoplasmic ratio, cell crowding and infiltration, round cell appearance, nesting, absent nucleoli, and syncytial growth pattern, among others [12].

## Case presentation

A 64-year-old man presented to his primary care provider with the chief complaint of passing small blood clots with urinary voiding. He stated that he had urinary frequency for three to four months before the hematuria, and he was also only able to void small volumes. He denied flank pain but endorsed a history of chronic lower back pain, attributed to the nature of his occupation as a general contractor. The patient additionally denied any personal history of kidney stones or family history of kidney, bladder, or prostate cancer. His mother died from complications of metastatic breast cancer (subtype unknown). He quit smoking 25 years prior to the presentation but was previously a one-pack-per-day smoker for 20-25 years. His urinalysis at presentation revealed >100 RBCs and trace leukocyte esterase with a negative corresponding culture result. His blood urea nitrogen (BUN) and creatinine levels were 27 and 1.93 mg/dL, respectively. His PSA was measured to be 4.32 ng/mL. These laboratory values in conjunction with the patient’s symptoms prompted urology referral.

**Figure 1:**
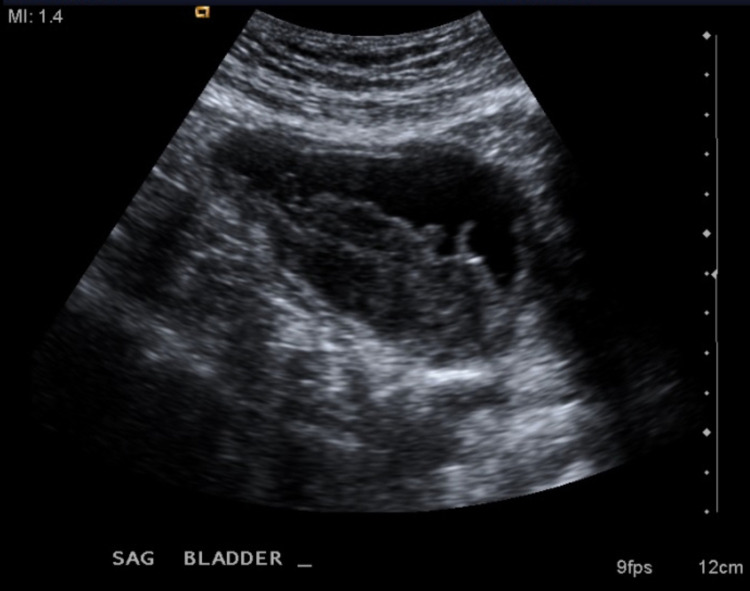
Ultrasound Findings This sagittal view of the bladder reveals a mass measuring 8.4 × 3.1 × 5.4 cm^3^ with observance of blood flow.

**Figure 2:**
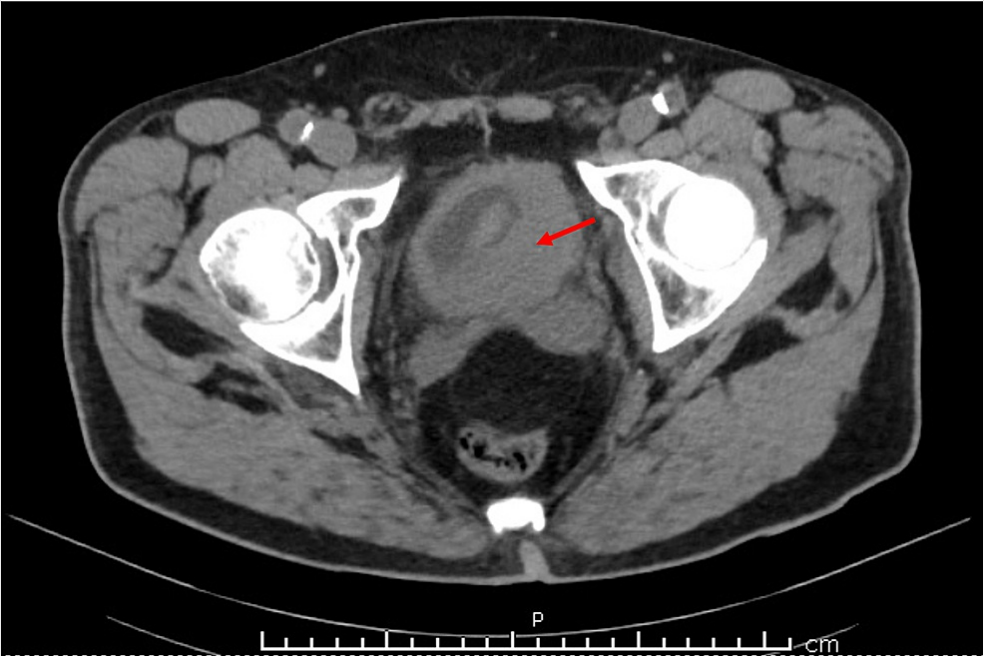
Transverse CT Findings This non-contrast CT scan from a transverse perspective reveals a tumor occupying a large portion of the bladder (highlighted by red arrow). Left-sided lymphadenopathy and vesicular fat infiltration are also visualized.

**Figure 3:**
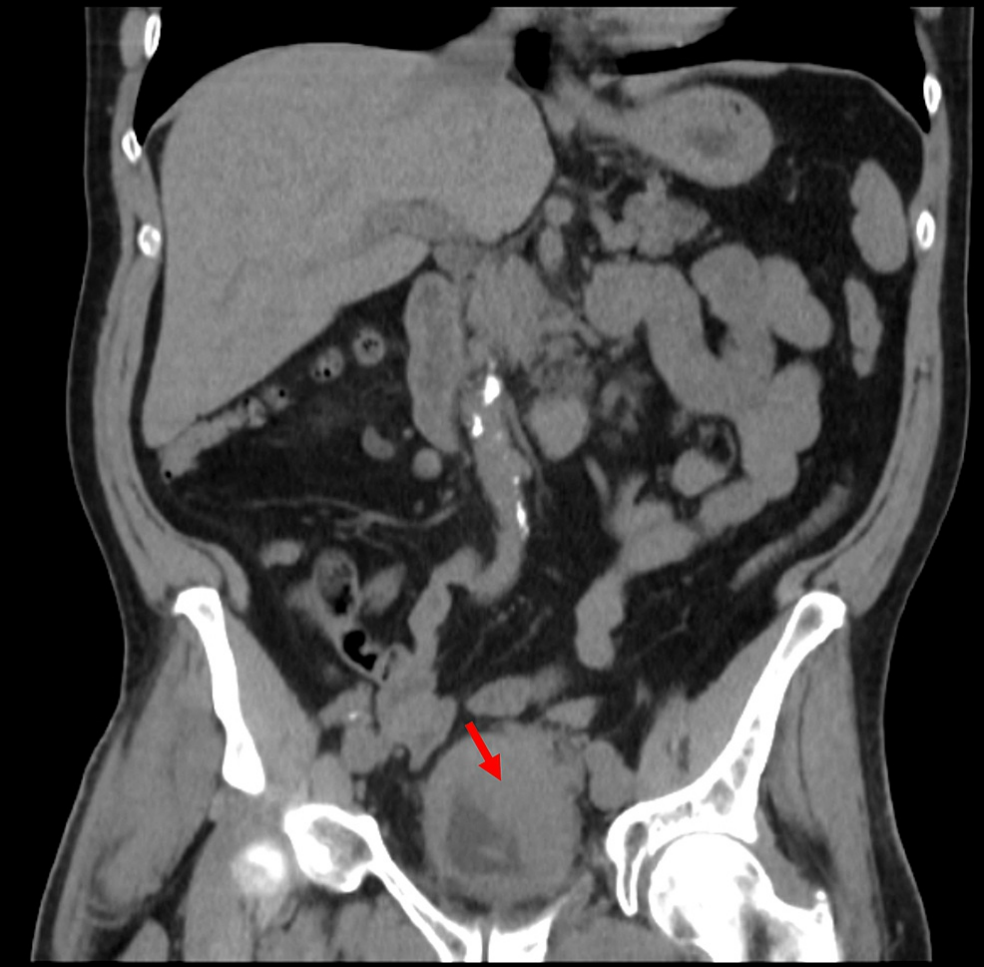
Coronal CT Findings This non-contrast CT scan from a coronal perspective reveals an infiltrating left-sided bladder tumor (highlighted by red arrow), lymphadenopathy, and fat infiltration.

**Figure 4:**
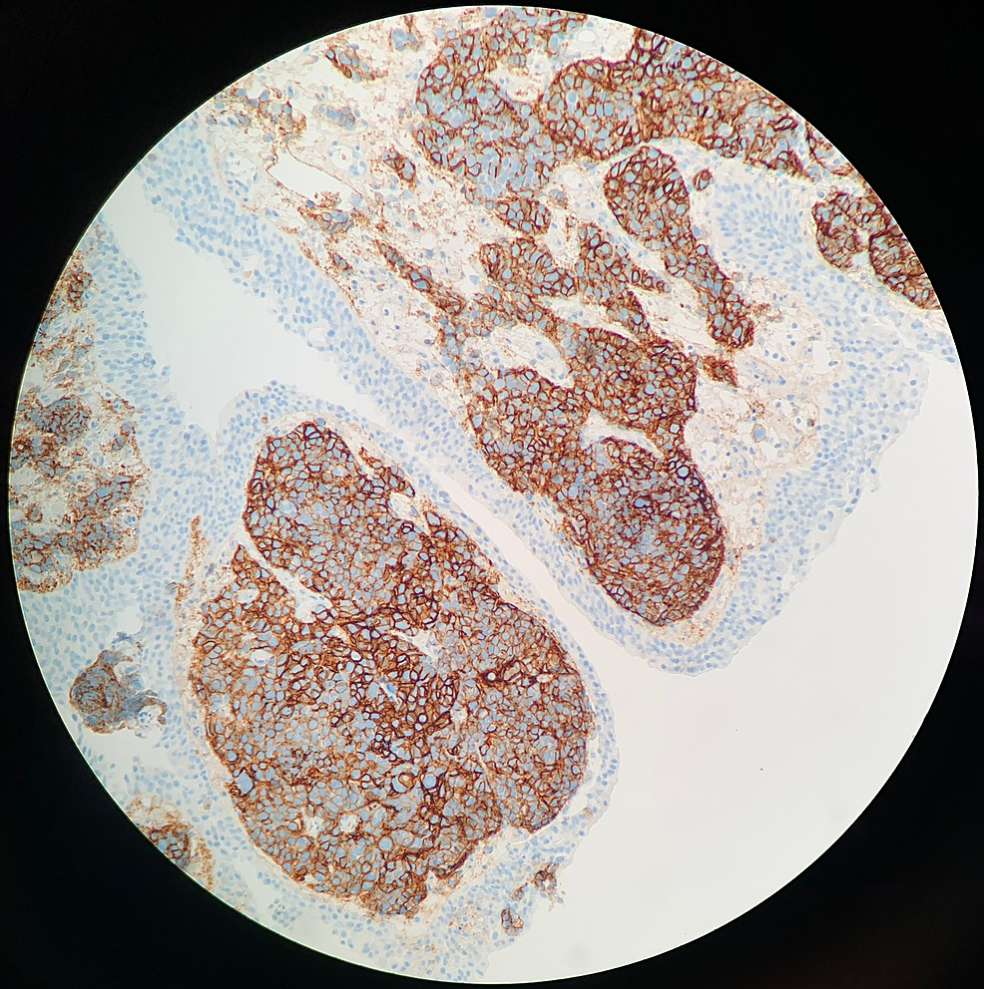
CD56 Positive Stain Immunohistochemical staining of the excised tumor biopsy shows diffusely positive CD56 staining (brown). This is a neuroendocrine marker that is often positive in small cell carcinoma [13].

**Figure 5:**
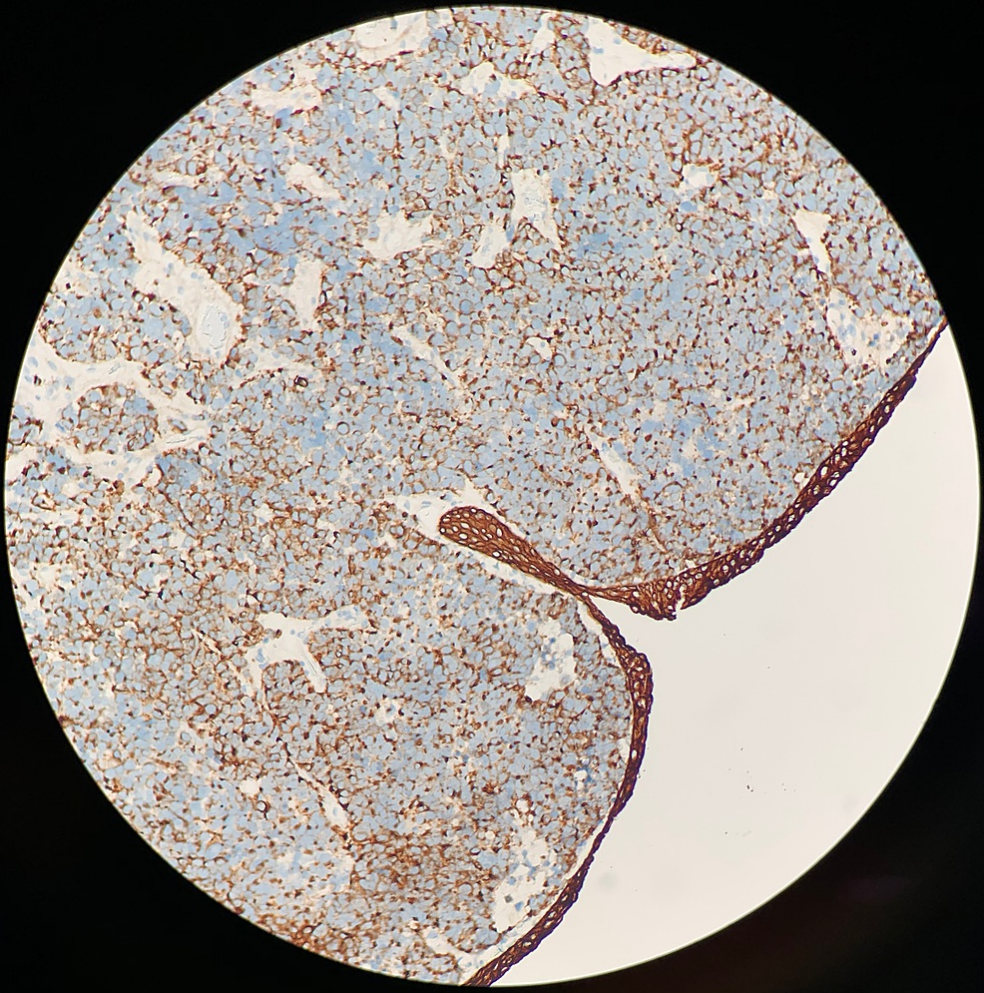
Cytokeratin (CK AE1/AE3) Positive Stain Immunohistochemical staining of the excised tumor biopsy shows diffusely positive cytokeratin or CK AE1/AE3 stain (brown). CK AE1/AE3 stains most carcinomas and epithelial tissue [14].

**Figure 6:**
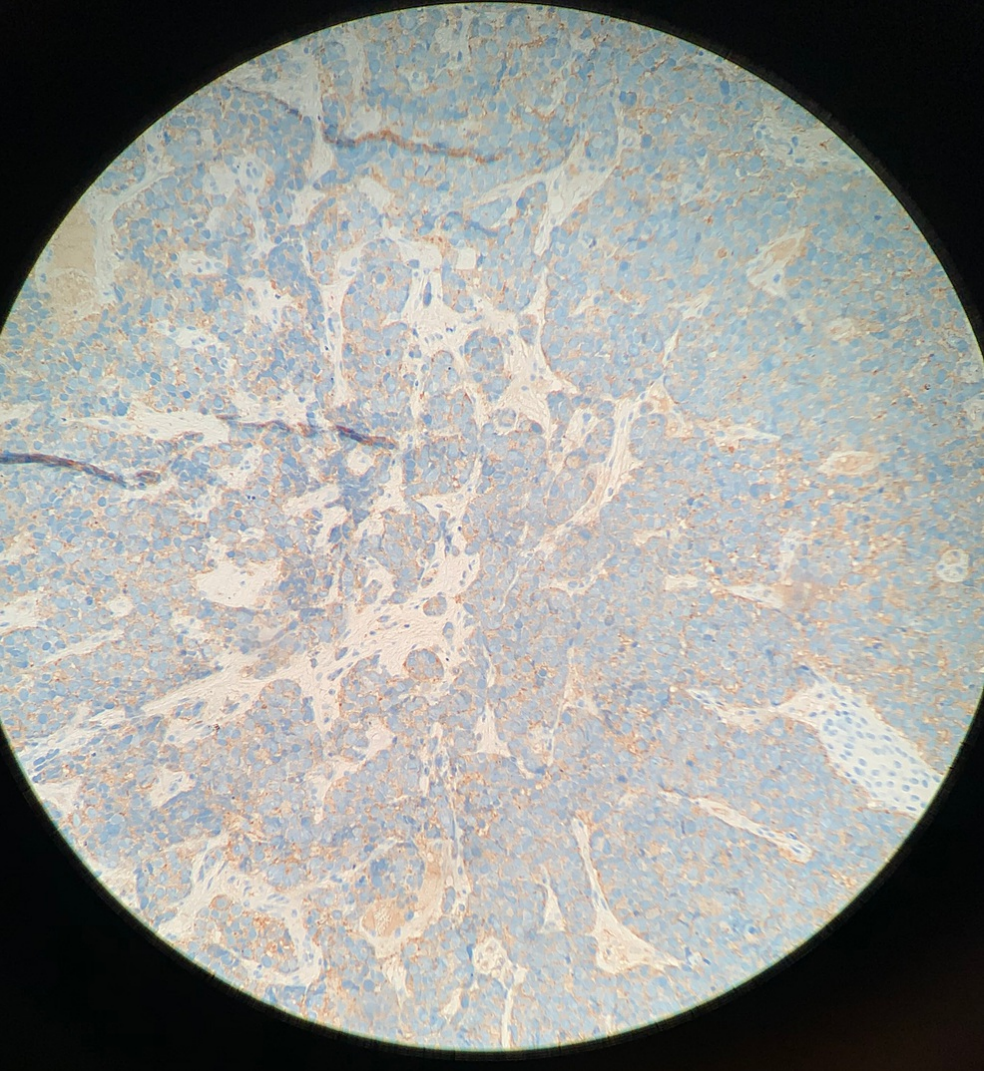
Synaptophysin Positive Stain Immunohistochemical staining of the excised tumor biopsy shows diffusely positive synaptophysin stain (blue). Synaptophysin is an integral membrane glycoprotein and it is commonly expressed in neuroendocrine tumors [15].

**Figure 7:**
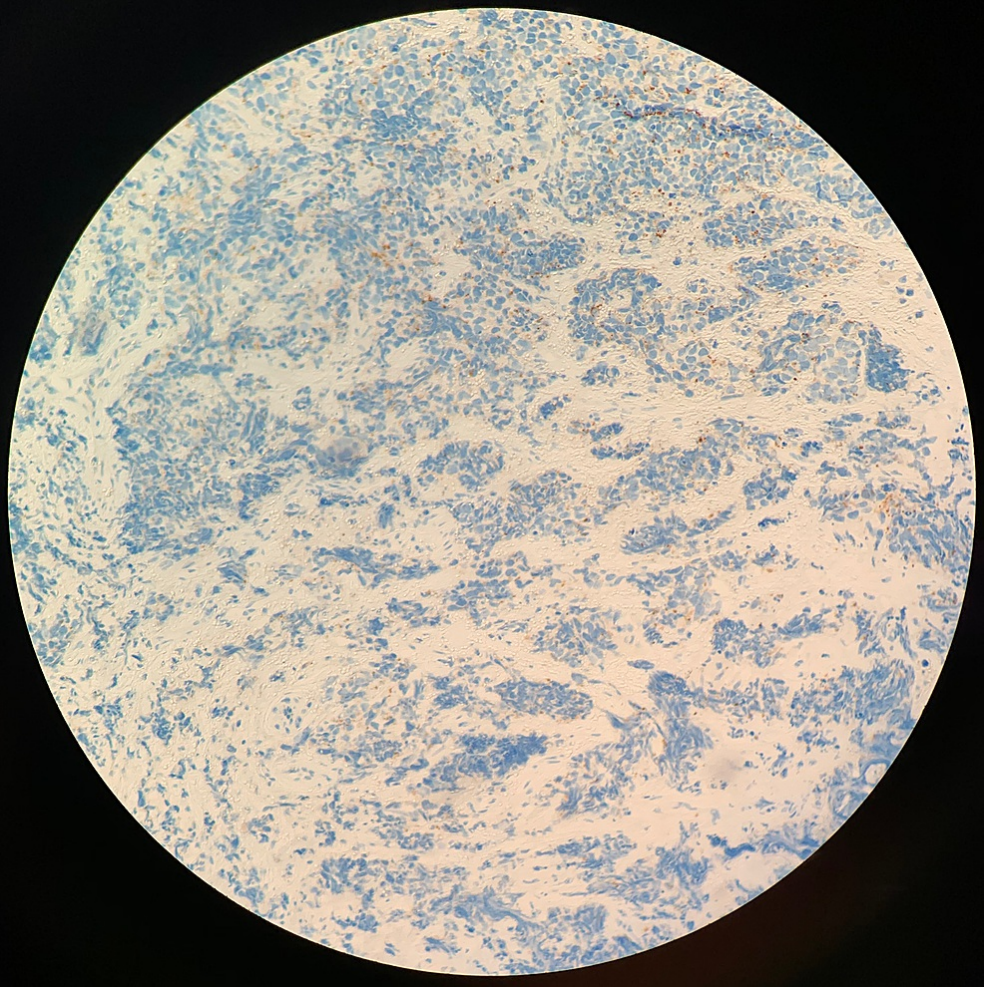
Chromogranin Weakly Positive Stain Immunohistochemical staining of the excised tumor biopsy shows a weakly positive chromogranin stain (blue). Chromogranin is a biomarker for neuroendocrine tumors [16].

**Figure 8:**
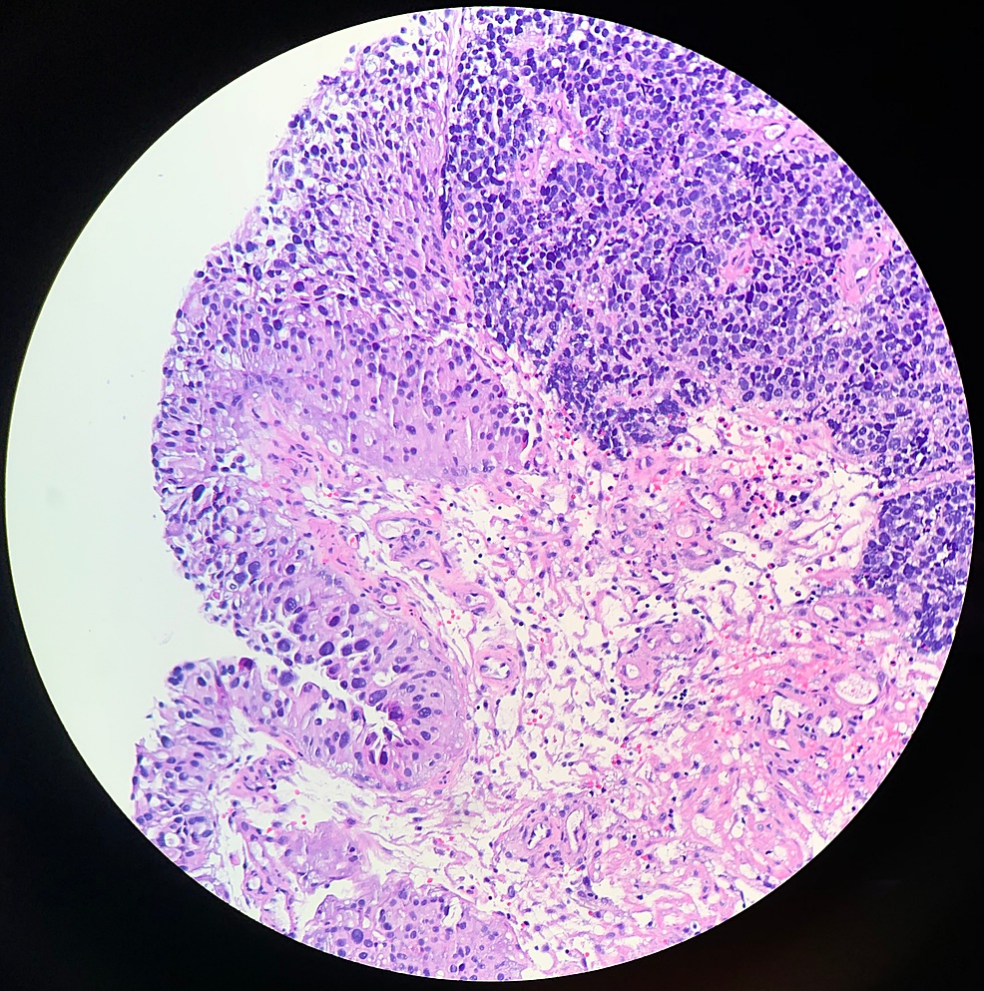
Small Cell Carcinoma and Urothelial Carcinoma In Situ H&E staining in conjunction with light microscopy visualization of the excised tumor biopsy shows both small cell carcinoma (top right) and urothelial carcinoma in situ (left) of the bladder. The small cell carcinoma displays classic features such as a high cellular nucleus to cytoplasm ratio, round cellular appearance, nesting, and crowding. The urothelial carcinoma in situ is not only limited to the transitional epithelial layer but also carries features of large nuclei and cell crowding. It is not yet infiltrating into the deeper layers of the bladder, as seen with the small cell carcinoma.

**Figure 9:**
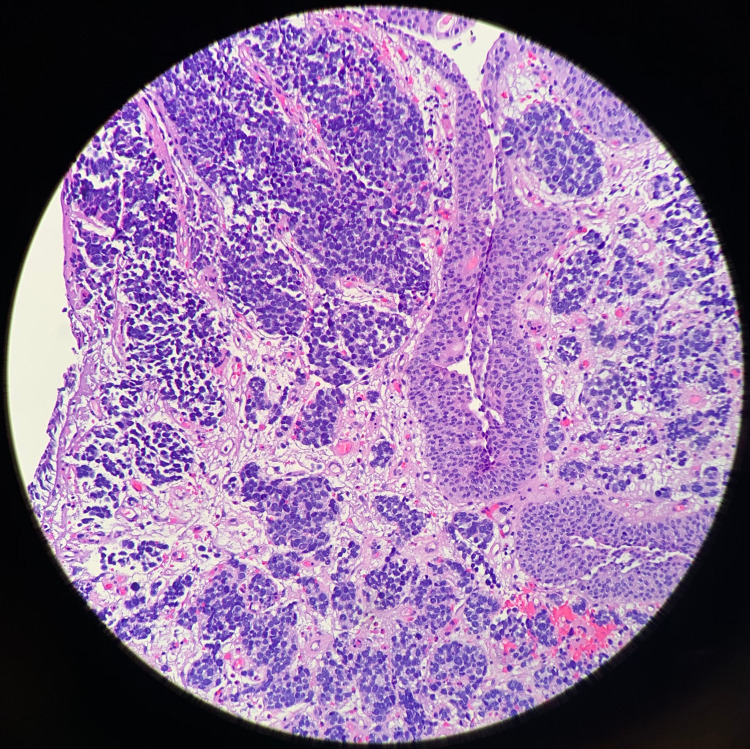
Small Cell Carcinoma H&E staining in conjunction with light microscopy visualization of the excised tumor biopsy shows the infiltrative nature of the small cell carcinoma (top left). The cells are within each layer of the bladder. The vascularity of the tumor is also demonstrated in this slide, especially in the bottom right.

Pending input from the urology team, a renal/bladder ultrasound (US) was ordered by the primary care provider in lieu of a CT with contrast due to the elevated kidney function tests. The US revealed a simple cyst of the right kidney and a large bladder mass measuring 8.4 × 3.1 × 5.4 cm^3^ with the observance of blood flow, which was suspicious for bladder carcinoma. The mass is shown in Figure 1. Bilateral hydronephrosis and a post-void bladder volume of 95 mL were additional concerning findings. After consultation from the urology team, the patient underwent a CT scan without contrast. The CT scan revealed left ureteral obstruction, a nodular outgrowth of the left aspect of the bladder with adjacent fat infiltration and nodularity. Left pelvic sidewall lymphadenopathy was also present. Figures 2 and 3 reflect these findings. A biopsy and potential resection of the tumor were performed subsequently via a transurethral resection of bladder tumor (TURBT) procedure to establish the diagnosis. Resection was not attempted at the urologist’s discretion due to the friability of the tissue. Pathology results revealed primary SCCB as suggested by diffuse positive CD56, CK AE1/AE3, and synaptophysin tissue staining. The tissue was weakly positive for chromogranin and negative for GATA-3. These positive markers are indicative of a tumor with a cellular subtype of neuroendocrine origin. The staining results are shown in Figures 4-7. Co-occurrence of UC in situ was also noted in the pathology report, further supporting the diagnosis of a primary SCCB. The two tumor subtypes are shown in Figures 8 and 9.

The patient was ultimately referred to an outside facility after establishing the diagnosis of SCCB. At this facility, a PET scan revealed extensive nodal, hepatic, and osseous metastases. There was no lung involvement. His cancer staging was determined to be T4N2M1. The patient completed targeted palliative radiotherapy (30 Gy/10 fractions over two to three weeks), which resulted in temporarily decreased hematuria. The chemotherapy regimen consisted of cisplatin, etoposide, and atezolizumab. These agents are commonly used in treating small cell carcinoma of the lung. The combination of these drugs and pain control with opiate medications resulted in several episodes of acute kidney injury and constipation. The patient also underwent the placement of bilateral nephrostomy tubes. After considering the nature of the disease, he discussed his advanced directives and underwent hospice care. The patient died six months after the initial presentation.

## Discussion

Bladder cancer classically presents as painless gross hematuria, which prompts further investigation and guideline-based workup with advisory from the National Comprehensive Cancer Network (NCCN) [17]. A differential diagnosis for hematuria may commonly include nephrolithiasis, acute kidney injury, trauma, dehydration, malignancy, and infection, among many others. Gross hematuria will typically prompt medical workup to include urinalysis, imaging, blood work, and a cystoscopy with biopsy to assist with diagnosis [17,18].

Common modifiable risk factors for bladder cancer include smoking history, exposure to arsenic in high concentrations in drinking water, occupational exposure (aromatic amines, hair dyes, and motor vehicle exhaust), among others [19,20]. Smokers have a four to seven times greater risk of developing bladder cancer than non-smokers [18,19]. Additional unmodifiable risk factors include male sex, Caucasian race, personal or family history of urinary tract cancer, and age greater than 55 years [21]. This case presentation is unique because the patient claimed initial symptoms of urinary frequency and low-volume voiding for three to four months before experiencing gross hematuria. It is unknown if he ever had documented microscopic hematuria. These primary symptoms are due to the tumor's physical size within the bladder and the inability to store large volumes of urine because of the space occupancy. The tumor measures at 8.4 × 3.1 × 5.4 cm^3^ on initial ultrasound, leaving little urine storage space. Figures 1-3 also demonstrate the space occupancy of the tumor. Additionally, the patient had almost all of the unmodifiable risk factors of bladder cancer including male sex, Caucasian race, and age greater than 55 years. However, he did not claim a personal or family history of kidney or bladder cancer. It is unknown whether the patient's mother's diagnosis of metastatic breast cancer is a contributory risk factor. With respect to SCCB, both the modifiable and unmodifiable risk factors are thought to be the same or similar to UC [7].

SCCB accounts for less than 0.7% of bladder cancers and fewer than 1,000 cases have been identified since 2017 [5,7,8]. It also commonly co-occurs with other bladder cancer subtypes, especially UC [8-10]. A 2015 study states just 600 cases have been identified with more than 60% being metastatic at diagnosis [6]. The most common sites for SCCB metastases are pelvic and retroperitoneal lymph nodes, liver, bone, brain, and lung. Approximately, 95% of SCCB cases are diagnosed at the T2 stage or higher, indicating muscle invasion [7]. For comparison, in 2010, there were 70,000 confirmed new cases of UC [1] and about 70-80% of cases are diagnosed in the non-muscle invasive stage (Stage Ta or T1) [18].

Because of its novelty, rarity, and aggressive nature, a standard treatment regimen for SCCB does not exist; however, according to the NCCN, all small cell carcinomas should all follow the same treatment guidelines as small cell carcinoma of the lung [17]. It should be noted that less than 4% of small cell carcinomas develop outside of the lung [8]. Any patient with small cell component histology with localized disease regardless of stage should complete chemoradiotherapy or neoadjuvant therapy, should the patient elect this route of care [17]. If the disease is localized, cystectomy is also recommended [17,22]. Neoadjuvant chemotherapy is considered the treatment of choice over surgical resection alone [22]. Adjuvant chemotherapy was assessed in the context after radical cystectomy in one study and found to improve mortality [23].

Due to the metastatic nature of the disease, this patient was not a candidate for cystectomy. Due to tumor friability during the TURBT procedure, he was also not a candidate for debulking. Future efforts should involve the compilation of social histories from each identified patient with the disease. This additional information allows for outcome reporting for treatment modalities and would increase the scientific knowledge base. To further explore the “clone origin” theory, documentation of other subtypes of bladder cancer along with SCCB should be encouraged [8-10]. Greater knowledge of this theory may lead to better, more targeted treatment modalities.

The patient’s most significant complaint was gross hematuria and the passage of blood clots, which prompted the course of palliative radiotherapy. This treatment decreased the frequency and volume of hematuria and provided him with better quality of life temporarily. The nephrostomy tube placement also aligned with care goals, which involved urinating without pain and obstruction. Pain management was also a critical component of this case, which involved opioid medications and appropriate bowel regimens to avoid constipation. In the case of any patient with metastatic disease, it is important to assess the patient’s insight and knowledge of the outcome, construct a goal-oriented care plan, and provide the patient with honor and dignity in the last moments of life. Holistic patient assessment is essential to achieve the best quality of life.

## Conclusions

Small cell carcinoma of the bladder accounts for an estimated 0.7% of all bladder cancers in contrast to UC, which accounts for 90% of bladder cancers. Current research and knowledge of the disease indicate similar risk factors between SCCB and UC including but not limited to cigarette smoking history, environmental exposures, male sex, Caucasian race, age >55 years, and personal/family history of cancer. Greater than 60% of SCCB cases are metastatic, in contrast to UC in which 70-80% of cases present in the Ta or T1 stages (without bladder muscle invasion). SCCB commonly co-occurs with other subtypes of bladder cancers, most notably UC (seen in 50-74% of cases). This suggests that the two cancer subtypes may have the same precursor cell type. Further exploration into this “clone origin” theory is encouraged and may lead to better, more targeted treatment modalities. At this time, the NCCN recommends that all carcinomas with a small cell component be treated equivalently to small cell carcinoma of the lung, which accounts for 96% of all small cell carcinomas. Cystectomy is also recommended in patients with localized disease.
